# Radioiodine-Refractory Thyroid Cancer: Molecular Basis of Redifferentiation Therapies, Management, and Novel Therapies

**DOI:** 10.3390/cancers11091382

**Published:** 2019-09-17

**Authors:** Mohamed Aashiq, Deborah A. Silverman, Shorook Na’ara, Hideaki Takahashi, Moran Amit

**Affiliations:** 1 Department of Head and Neck Surgery, Division of Surgery, The University of Texas MD Anderson Cancer Center, Houston, TX 77030, USA; MOHAMedaashiq@gmail.com (M.A.); htk98@yokohama-cu.ac.jp (H.T.); 2 Department of Melanoma Medical Oncology, Division of Cancer Medicine, The University of Texas MD Anderson Cancer Center, Houston, TX 77030, USA; DASilverman@mdanderson.org; 3 Department of Otolaryngology, Head and Neck Surgery, and the Laboratory for Applied Cancer Research, Rappaport Institute of Medicine and Research, The Technion, Israel Institute of Technology, Haifa 3109601, Israel; N_SHOROOK@rambam.health.gov.il

**Keywords:** radioactive iodine-refractory, differentiated thyroid cancer, papillary thyroid cancer, tyrosine kinase inhibitor, sodium/iodide symporter, braf

## Abstract

Recurrent, metastatic disease represents the most frequent cause of death for patients with thyroid cancer, and radioactive iodine (RAI) remains a mainstay of therapy for these patients. Unfortunately, many thyroid cancer patients have tumors that no longer trap iodine, and hence are refractory to RAI, heralding a poor prognosis. RAI-refractory (RAI-R) cancer cells result from the loss of thyroid differentiation features, such as iodide uptake and organification. This loss of differentiation features correlates with the degree of mitogen-activated protein kinase (MAPK) activation, which is higher in tumors with *BRAF* (B-Raf proto-oncogene) mutations than in those with *RTK* (receptor tyrosine kinase) or *RAS* (rat sarcoma) mutations. Hence, inhibition of the mitogen-activated protein kinase kinase-1 and -2 (MEK-1 and -2) downstream of RAF (rapidly accelerated fibrosarcoma) could sensitize RAI refractivity in thyroid cancer. However, a significant hurdle is the development of secondary tumor resistance (escape mechanisms) to these drugs through upregulation of tyrosine kinase receptors or another alternative signaling pathway. The sodium iodide symporter (NIS) is a plasma membrane glycoprotein, a member of solute carrier family 5A (*SLC5A5*), located on the basolateral surfaces of the thyroid follicular epithelial cells, which mediates active iodide transport into thyroid follicular cells. The mechanisms responsible for NIS loss of function in RAI-R thyroid cancer remains unclear. In a study of patients with recurrent thyroid cancer, expression levels of specific ribosomal machinery—namely PIGU (phosphatidylinositol glycan anchor biosynthesis class U), a subunit of the GPI (glycosylphosphatidylinositol transamidase complex—correlated with RAI avidity in radioiodine scanning, NIS levels, and biochemical response to RAI treatment. Here, we review the proposed mechanisms for RAI refractivity and the management of RAI-refractive metastatic, recurrent thyroid cancer. We also describe novel targeted systemic agents that are in use or under investigation for RAI-refractory disease, their mechanisms of action, and their adverse events.

## 1. Introduction

Radioactive iodine I-131 (RAI) is a cornerstone in the routine adjuvant management in patients with high-risk differentiated thyroid cancer (DTC) [1]; however, 5% to 15% of DTC and 50% of metastatic DTCs are refractory to RAI treatment [2,3,4]. Patients with RAI-refractory (RAI-R) thyroid cancer have poor outcomes, with 5-year disease-specific survival rates of 60% to 70% [5]. Those with RAI-R metastatic thyroid cancer have the worst outcomes, with a 10-year survival rate of 10% [6]. Hence, resensitizing RAI-R tumors to RAI can potentially improve survival for patients with DTC.

With recent advances and developments in understanding of the oncogenic pathways involved in the development of thyroid cancers and the molecular basis of RAI refractoriness, targeted therapies are being developed and are showing promising results [7,8,9,10]. Here, we review the molecular mechanisms underlying RAI refractoriness, describe targeted therapies that may overcome these mechanisms, and explore promising therapeutic regimens to improve outcomes in RAI-R thyroid cancers.

## 2. Definition of RAI-R Tumors

A major obstacle to standardizing the approach to RAI-R tumors is the lack of a consistent definition for RAI-R. The following definitions for RAI-R tumors are used in the literature [11,12,13]:

i. Absence of RAI uptake at initial diagnosis of locoregional recurrence or distant metastasis;
ii. Absence or progressive loss of radioiodine uptake in the post-therapy scan several days after RAI therapy;
iii. Presence of more than 1 metastatic lesion with at least one lesion without RAI uptake in the post-therapy scan;
iv. Structural progression of tumors 12 to 16 months after RAI therapy despite the presence of iodine uptake in the post-therapy scan;
v. Tumors in patients who have received 600 millicurie (mCi)/22.2 gigabecquerel (GBq) or more of RAI cumulatively without signs of remission;
vi Significant uptake on 2-deoxy-2-[fluorine-18] fluoro-D-glucose positron emission tomography integrated with computed tomography (F-18 FDG PET/CT).

None of the above criterion alone portends that a tumor is RAI-R, rather it predicts the likelihood that a tumor will be RAI-refractory and should be used together for risk stratification of the tumors in assessing for refractoriness. It is important for the RAI uptake scan to be standardized and an optimal approach is required for patient preparation and choice of imaging modality [14].

Aggressive or poorly differentiated tumors on histology or tumors exhibiting aggressive genetic profiles (such as *BRAF* and telomerase reverse transcriptase (*TERT*) promoter mutation) can also be included for risk stratification of patients [15].

## 3. Molecular Mechanisms Driving Primary RAI Refractoriness

**Figure 1:**
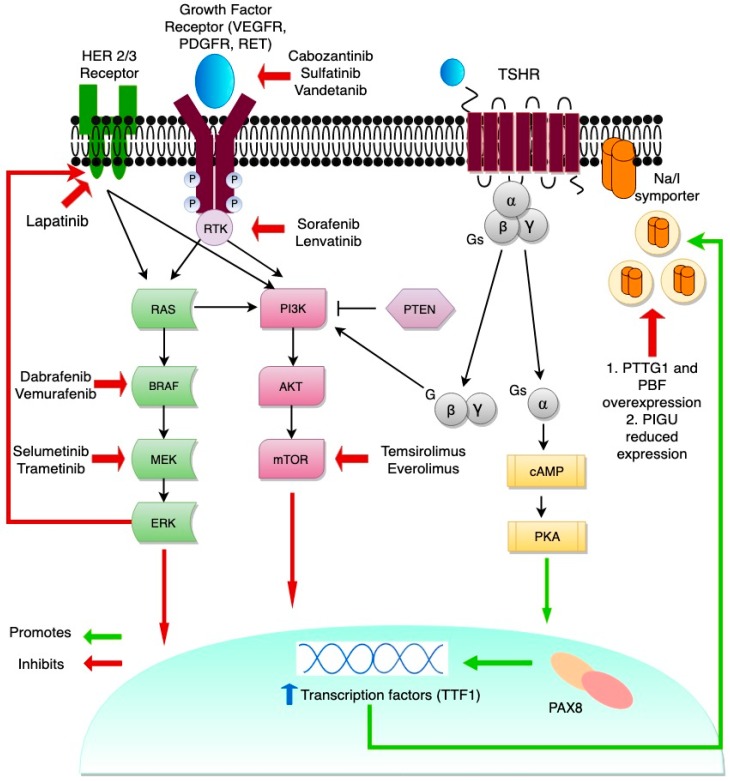
Redifferentiation of thyroid cancer. MAPK (mitogen-activated protein kinase) (RAS/RAF/MEK) and PI3K/AKT/mTOR are key signaling pathways in thyroid cancer pathogenesis. Signaling cascades can be blocked by new targeted therapies. The crosstalk between MAPK and PI3K through RAS is shown and represents a tumor escape mechanism from known multi-kinase inhibitors and selective inhibitors of BRAF. PI3K-AKT pathway activation leads to suppression of NIS (sodium/iodide symporter) glycosylation and surface translocation. The inhibition of mTOR promotes redifferentiation of thyroid cancer cells by upregulation of NIS mRNA and protein expression through increased transcription of TTF1. Another important positive regulator of NIS expression is PTEN. TSH (thyroid stimulating hormone) signals through the heterotrimeric G-protein complex, and through activation of cAMP increases transcription of the *NIS* gene. Aberrant activation of the MAPK signaling pathway inhibits PIGU expression and NIS basolateral transport. PTTG1 and PBF overexpression results in decreased NIS levels in thyroid cancer.

RAI refractoriness most frequently develops in the context of loss of thyroid differentiation features. One hallmark of dedifferentiation is impairment of Na/I symporter (NIS) function [16,17]. NIS is a plasma membrane glycoprotein, a member of solute carrier family 5A (*SLC5A5*), located on the basolateral surface of the thyroid follicular epithelial cells. NIS mediates active iodide transport into thyroid follicular cells, and in the normal thyroid cells, Thyroid stimulating hormone (TSH) stimulates NIS expression via the cyclic adenosine monophosphate (cAMP) pathway, binding to the NIS promoter via paired box 8 (PAX8) [18]. The ability of follicular cells to concentrate iodine is exploited in thyroid cancer therapy; RAI enters the cells via NIS and emits beta particles that destroy the follicular cell [16,19]. In a subset of DTCs, impaired targeting to the plasma membrane or impaired intracellular retention of NIS results in NIS loss, producing RAI resistance [20]. Figure 1 summarizes the transcriptional and translational pathways involved in regulation of NIS expression. By deconvoluting the mechanisms involved in NIS downregulation or loss of function in thyroid cancer, it is possible to identify targets that could potentially overcome RAI refractoriness in thyroid cancers [17,20].

Genetic and epigenetic alterations in the RTK/BRAF/MAPK/ERK and PI3K-AKT-mTOR pathways by acquired point mutations, chromosomal rearrangement, or aberrant gene methylation underly the diminished NIS signaling central to RAI refractoriness [21,22,23]. The most well-defined example of a point mutation aberrantly activating these signaling pathways is the activating hotspot *BRAF^V600E^* mutation. *BRAF^V600E^* represents the most frequent genetic aberration in thyroid cancers, occurring in nearly 50% of DTCs. *BRAF^V600E^* mutations both inversely correlate with NIS expression and directly correlate with dedifferentiation, recurrence, and metastasis [24,25]. BRAF activation has been found to repress NIS expression via two defined pathways. First, BRAF activates TGFβ (Transforming growth factor β)/Smad3 signaling, which directly impairs the ability of the thyroid-specific transcription factor PAX8 (paired box gene 8) to bind the *NIS* promoter in follicular cells [26]. Second, BRAF epigenetically regulates *NIS* by driving histone deacetylation of the H3 and H4 lysine residues of the *NIS* promoter, directly preventing its transcription [27].

## 4. Management of RAI-R Thyroid Cancers

### 4.1. Monitoring and Watchful Waiting

RAI-R metastatic DTCs can be asymptomatic for several years [28]. Active surveillance and watchful waiting with TSH suppression can be employed in patients with asymptomatic disease, low tumor burden or tumor size less than 1 cm, and minimally progressive tumors [12,13,29,30]. Presence of small (<8 mm) and asymptomatic metastatic lymph nodes after RAI therapy with previous neck compartmental dissection and small (<1 cm) metastatic pulmonary nodules can be followed up for years with neck ultrasonography and axial imaging for the pulmonary nodules. Other imaging modalities, such as F-18 FDG PET/CT, and thyroglobulin levels in TSH-suppressed patients can also be used to assess disease progression [12,13,31,32,33]; these are used in conjunction with axial imaging when growth of lesions is suspected [34].

### 4.2. Local Therapy

In locoregional relapse, surgery is still the most commonly used therapeutic approach, with therapeutic compartmental central or lateral neck dissection to spare uninvolved vital structures, or a more limited surgery in cases of prior comprehensive neck dissection [12,13]. External-beam radiation therapy (EBRT) is commonly used alone or in combination with surgery in bone and central nervous system (CNS) metastasis of thyroid cancers [35,36]. Some studies have demonstrated benefit in locoregional control and good prognosis with surgery combined with EBRT in doses of 40 to 50 Gy in patients 45 years and older [37]. Limited outcome data are available on other locoregional therapies, such as radiofrequency ablation, and ethanol ablation or embolization [13]. It is of note that symptomatic patients with metastatic lung nodules or bone lesions are usually considered for local therapies before systemic therapies [38].

### 4.3. Targeted Therapies Using Tyrosine Kinase Inhibitors

Targeted treatments for thyroid cancer have been increasingly developed over the last decade along with increasing knowledge about the disease’s underlying molecular alterations. Most agents that were tested in phase II and III trials were developed for treatment of advanced RAI-R thyroid cancer. 

Cellular dedifferentiation in thyroid cancers causes tumor progression in the form of more aggressive growth, metastasis, loss of iodide uptake, or unresponsiveness to RAI therapy, and correlates with the degree of MAPK activation. Tyrosine kinases are involved in the MAPK signaling pathway through phosphorylation/dephosphorylation of several intracellular proteins, which underlies the rationale for the use of tyrosine kinase inhibitors (TKIs) in the treatment of thyroid cancer [21,39].

TKIs have been shown to significantly improve progression-free survival rates in advanced RAI-R DTCs. Overall survival has been difficult to document in these trials, likely because patients cross over to the drug arm once disease progression is documented in the control group [9,10]. An overall survival benefit was observed with the use of lenvatinib in selected patients > 65 years of age with RAI-R DTCs [40]. Two TKIs, lenvatinib and sorafenib, are currently used for the treatment of RAI-R DTC (Table 1), and two others, vandetanib (NCT01876784) and cabozantinib (NCT03690388), are under investigation in phase III trials for patients with progressive RAI-R DTCs and advanced RAI-R DTCs unresponsive to previous VEGFR therapy, respectively [41,42]. 

Although TKIs have revolutionized the field of targeted therapy in RAI-R DTC patients, these agents are usually administered lifelong, and several drawbacks were associated with their long-term application. TKIs’ adverse effect profiles have a great impact on quality of life and should be taken into consideration by the clinician prior to treatment initiation. Also, a resistance to the treatment, also called “escape”, can develop; hence, access and adherence to close monitoring with a continuous assessment of adverse effects and the patient’s quality of life should be considered in the decision to start the therapy [43]. In clinical settings with no preselection of patients, lenvatinib has been shown to be useful in the management of RAI-R DTCs with implementation of good and specified management protocols for toxicities and adverse events [44,45]. 

Potential targets are also transcriptional factors such as vascular endothelial growth factor (VEGF). In response to intratumoral hypoxia, hypoxia inducible factor-1 alpha (HIF-1α) is activated and induces VEGF transcription together with co-stimulation by growth factor signaling pathways, such as the PI3K/AKT and MAPK pathways [46,47]. VEGF is a promoter of angiogenesis and is an attractive target for therapy. Another target for HIF-1α is the MET oncogene, which is overexpressed in thyroid cancers, especially medullary thyroid cancer (MTC), thereby promoting angiogenesis, cellular motility, invasion, and metastasis [48,49].

### 4.4. Tumoral Escape Mechanisms from Targeted Therapies

As discussed above, thyroid cancers often become RAI-R by co-opting RAF and RAS signaling, thereby repressing NIS and RAI uptake. Although targeted therapies such as BRAF inhibitors have shown some success in resensitizing tumors to RAI, these tumors often escape RAI sensitivity via aberrations in complementary pathways. In patients with documented response to targeted therapies, after several months, the tumor escapes (i.e., it ceases to respond and starts growing again). Several hypotheses and suggested mechanisms have been proposed to explain such escape, most of which involve overactivation of alternative pathways to overcome the drug’s effect [50,51].

Combining adjuvant therapies with TKIs has the potential to eliminate or delay the escape effect (i.e., resistance) and result in longer progression-free survival. Numerous genetic and signal transduction alterations have been observed in RAI-R or advanced papillary thyroid cancer (PTC) [15,21,23], and simultaneous targeting of these alterations might allow more durable tumor control when combined with current TKIs.

One unique mechanism by which thyroid cancer drives RAI resistance may be via upregulation of the human epidermal receptor (HER) family of receptor tyrosine kinases, which is present in more than one-third of thyroid cancers and is positively correlated with local tumor invasiveness [52]. HER2 and HER3 are key players upstream of extracellular signal-regulated kinase (ERK) and AKT, and HER2 and HER3 activate these signaling pathways. Interestingly, overexpression of HER2 and HER3 may provide a mechanism of RAI-R tumor escape for *BRAF* mutant cells treated with the BRAF inhibitor vemurafenib [53]. Therefore, the addition of the HER2 inhibitor trastuzumab to vemurafenib treatment may enable patients with RAI-R tumors to overcome the escape phenomenon and experience a more durable effect from the targeted therapy [54].

Anaplastic lymphoma kinase (ALK) is a recently identified kinase with the potential to contribute to aggressive disease in non-small-cell lung cancers. This kinase can undergo rearrangements—the most common is EML4-ALK fusion with echinoderm microtubule–associated protein-like 4 (*EML4*) gene—that were described in a subset of patients, who tend to be younger with more aggressive disease [55]. ALK fusion proteins are known to activate various signaling pathways, such as the PI3K/AKT pathway and the MAPK pathways [56,57], and aberrant activation of these ALK fusion proteins promotes proliferation and survival in cancer cells [58]. ALK fusion has been demonstrated in medullary thyroid cancer and anaplastic carcinoma [59]. In RAI-R DTC, EML4-ALK fusion and several other ALK translocations were identified by whole genome sequencing [60]. A study of the translocation profile of ALK in DTC found ALK translocations in 11 of 498 papillary thyroid cancers (PTCs) (2.2%) and 3 of 23 diffuse sclerosing variant PTCs (13%). Combining specific ALK inhibitors such as crizotinib with standard adjuvant therapies might offer durable response in patients with ALK-positive tumors [61].

Alterations in the PI3K/AKT/mTOR cascade are well documented in thyroid cancer tumorigenesis (Figure 1). The inhibition of mTOR promotes redifferentiation of thyroid cancer cells by upregulating NIS mRNA and protein expression, resulting in elevated iodine uptake through increased transcription at the level of thyroid transcription factor-1 (TTF1), which indicates TTF1 dependence for NIS expression [62,63]. There is an inverse relationship between platelet-derived growth factor receptor-alpha (PDGFR-α) activation and transcriptional activity of TTF1, with PDGFR-α blockade restoring NIS expression [64]. Another important positive regulator of NIS expression is PTEN, which is suppressed by oncogenic miR-21; antisense-miR-21 increases NIS expression [65]. Some clinical trials have evaluated the mTOR inhibitor everolimus and the combination of sorafenib and the mTOR inhibitor temsirolimus in the treatment of RAI-R thyroid cancer. However, these studies were not designed to evaluate the change in RAI uptake or the effectiveness of combined RAI therapy with the drugs [66,67]. Further clinical trials are needed to elucidate the role of mTOR inhibition in inducing radioiodine avidity.

Downstream mechanisms in the signaling pathways involved in RAI refractoriness affect the posttranslational modifications or shuttling of the transcribed NIS. Pituitary tumor transforming gene 1 (PTTG1) and PTTG-1 binding factor overexpression in thyroid cancers results in decreased NIS levels [68], likely through its retention in clathrin-coated vesicles or by repressing NIS mRNA transcription [69]. PI3K-AKT pathway activation in thyroid carcinoma leads to suppression of NIS glycosylation and surface translocation [70]. In a novel mechanism recently described by our group, decreased expression of ribosomal machinery subunits (i.e., phosphatidylinositol glycan anchor biosynthesis class U) resulted in improper NIS post-translational processing and deregulated trafficking of the protein to the plasma membrane, resulting in increased RAI refractoriness in thyroid cancer cells [71].

### 4.5. Other Systemic Therapies

Phase II trials of the efficacy of various chemotherapy agents for recurrent and metastatic DTC have been reported; in these, doxorubicin was the most frequently used agent [72]. No consensus has been reached for the use of a specific cytotoxic regimen in RAI-R disease, and clinical trials of cytotoxic chemotherapy, in addition to TKIs and other targeted therapies, are needed in patients with RAI-R disease [1].

### 4.6. Targeted Therapies and Tumor Immune Microenvironment in RAI-R Thyroid Cancer

Characterizing the immune landscape following TKI treatment in various tumor types, including thyroid cancer, has demonstrated dynamic alterations in the tumor immune microenvironment [73,74]. Using a TKI that targets the Vascular endothelial growth factor-A/Vascular Endothelial Growth Factor Receptor (VEGF-A/VEGFR) axis affects regulatory T cell percentages and seems to increase PD-1 (Programmed cell death protein 1) expression, which leads to inhibition of cytotoxic T cells [73]. Immune profiling of *BRAF-V600E*-positive DTC revealed high levels of PD-L1 (Programmed death-ligand 1) (53% vs. 12.5%) and human leukocyte antigen G (41% vs. 12.5%) compared with *BRAF* wild-type tumors. Furthermore, *BRAF-V600E*-positive tumors had a high level of suppressive T cell and macrophage components [74]. These results show the inhibitory effects of the aberrant tyrosine kinases on the immune system, indicating the potential of TKIs to reverse them, with the combination of TKIs and immune checkpoint inhibitors seemingly an attractive regimen for patients with RAI-R DTC. 

Ongoing trials are evaluating the role of TKIs in advanced RAI-R thyroid cancer, either alone or in combination with immune checkpoint inhibitors or RAI therapy [75]. Sulfatinib is an oral TKI targeting VEGFR, FGFR-1 (Fibroblast growth factor receptor 1), and CSF1R (colony-stimulating factor 1 receptor); therefore, it might play a dual antiangiogenic and immunomodulatory role. The early results of NCT02614495, an open label, two-cohort, phase I and II trial of sulfatinib in RAI-R DTC, were presented in 2017. In this trial, patients were assigned to sulfatinib. Partial responses were confirmed in 3 of 12 patients with DTC; all others achieved stable disease [76]. An ongoing phase IB/II trial (NCT02501096), is assessing the maximum tolerated dose of lenvatinib combined with the PD-1 inhibitor pembrolizumab in patients with solid tumors, including thyroid cancer [77]. Another ongoing trial, NCT01988896, is evaluating the PD-L1 inhibitor atezolizumab combined with the MAPK inhibitor cobimetinib in patients with locally advanced or metastatic solid tumors [78].

### 4.7. Current Recommendations for Treatment of Symptomatic RAI-R Thyroid Cancer

Current American Thyroid Association (ATA) guidelines recommend high-risk metastatic progressive (i.e., at least 20% increase in sum of longest diameter of lesions) RAI-R-DTCs not amenable to conventional therapies be considered for TKIs in specialized centers. Since immunotherapies and re-sensitization therapies are currently in the phase of clinical trials, the ATA recommends admittance into these trials if RAI-R DTCs are progressive after use of approved TKIs, such as lenvatinib or sorafenib. Molecular characterization of these lesions can help to identify and select the appropriate clinical trials. The ATA endorses the use of EBRT or radiofrequency ablation or cryoablation over surgery for symptomatic distant metastatic lesions or lesions with high risk of local complications prior to initiation of TKIs. It also advocates their use for single or multiple progressive lesions while on TKIs or other novel therapies [1]. 

## 5. Current and Future Perspectives with NIS Restoration in RAI-R Thyroid Cancer Redifferentiation

Novel therapies with single kinase inhibitors have been shown to re-induce iodide uptake in RAI-R thyroid cancer cells. The MEK 1/2 inhibitor selumetinib was found to reverse refractoriness to RAI in patients with advanced or metastatic DTC [8]. Rothenberg et al. reported that dabrafenib, a selective inhibitor of mutant BRAF, resulted in iodide re-uptake in patients with *BRAF-V600E*-positive RAI-R PTCs [79]. Sabra et al. showed that RAI therapy was ineffective against metastatic, RAS-mutated, RAI-avid FTC, but pretreatment with an MAPK inhibitor improved responsiveness to RAI therapy [39]. A major advantage of this treatment with a single kinase inhibitor is its shorter treatment duration (45 days) compared with long-term administration in TKIs; thus, associated adverse effects and the development of resistance are minimized [13,80].

BRAF inhibitors (e.g., dabrafenib) and MEK inhibitors (e.g., selumetinib) activate PI3K and MAP-K pathways by inducing HER3 gene expression. The HER3 inhibitor lapatinib prevents this MAPK rebound and sensitizes *BRAF-V600E*-positive thyroid cancer cells to RAF or MAP/ERK inhibitors [53]. Use of a HER inhibitor in combination with a BRAF/MEK inhibitor increased sensitivity of *BRAF-V600E*-positive PTC to a BRAF/MEK inhibitor by preventing MAPK rebound and increased NIS expression [81]. These findings show that HER3 signaling is accompanied by an increase in ERK in the MAPK pathway and is a possible target for treating RAI-R thyroid cancer. Currently, a phase I study (NCT01947023) is evaluating combination therapy with dabrafenib and lapatinib in patients with *BRAF* mutated RAI-R DTC [82].

ERK rebound could be inactivated in a sustained manner with the use of a MEK inhibitor and by thwarting RAF reactivation in *BRAF*-mutated thyroid cancer, resulting in restoration of RAI uptake through increased NIS expression [83]. This concept is currently being evaluated in a phase II clinical trial (NCT03244956) using combination of Trametinib (MEK inhibitor) and Dabrafenib (BRAF inhibitor) in RAI-R DTC patients with two independent arms of *RAS* and *BRAFV600E* mutations [84].

## 6. Conclusions

Efficacy and toxicity should be considered in the choice of agents to treat RAI-R thyroid cancer. These agents include axitinib, cabozantinib, pazopanib, sorafenib, sunitinib, and vandetanib, including the selective BRAF inhibitors vemurafenib and dabrafenib (Table 2). 

RAF and MEK inhibitors have shown promise in the redifferentiation of RAI-R DTC; verification of these results could improve treatment options for patients with RAI-R DTC. New targeted agents, immune checkpoint inhibitors, and combinations of agents for redifferentiation show promise and could improve the efficacy of RAI therapy for patients with RAI-R thyroid cancer.

## Figures and Tables

**Table 1:** Completed Phase III Clinical Trials of Agents Approved for the Treatment of Differentiated Thyroid Cancer by the U.S. Food and Drug Administration [9,10].

*Parameters*	DECISION Trial: Sorafenib	SELECT Trial: Lenvatinib
Drug targets	Specific target: RAF Other targets: VEGFR, c-Kit, RET, PDGFR, FLT3	Specific target: FGFR Other targets: VEGFR, c-Kit, RET, PDGFR, RET-KIF5B, CCDC6-RET, NcoA4-RET rearrangement
Patient population	*N* = 417, randomized 1:1 dose: 800 mg daily	*N* = 392, randomized 2:1 dose: 24 mg daily
Median progression-free survival (months)	10.8 vs. 5.8 (*p* < 0.0001)	18.3 vs. 3.6 (*p* < 0.001)
Complete response	0% vs. 0%	1.5% vs. 0%
Partial response	12.2% vs. 0.5%	63.2% vs. 1.5%
Stable disease > 23 weeks	41.8% vs. 33.2%	15.3% vs. 29.8%
Grade 3 and 4 adverse effects	Overall: 37.2% vs. 26.3%	Overall: 75.9% vs. 9.9%
	Hand-foot syndrome: 20.3%	Hypertension: 42%
	Hypertension: 9.7%	Proteinuria: 10%
	Hypocalcemia: 5.8%	Thromboembolic effects: 6.5% (arterial vs. venous: 2.7% vs. 3.8%)
	Weight loss: 5.8%	Acute Renal failure: 1.9%
	Diarrhea, fatigue: 5.3%	QT prolongation: 1.5%
	Rash/desquamation: 4.8%	Hepatic failure: 0.4%
	Shortness of breath: 4.8%	
Dose reduction	64.3%	67%
Treatment discontinuation	19%	14%

**Table 2:** Phase I and II Trials of Multi-Kinase Inhibitors for RAI-R Thyroid Cancers.

Drug	Drug Targets	Phase	Type of Thyroid Cancer	Response Rate (Complete or Partial Response)	Median Progression Free Survival (Months)
Axitinib (Locati et al. [85])	VEGFR, PDGFR, c-Kit	II	Advanced DTC, MTC	35%	16.1
Axitinib (Cohen et al. [86])	VEGFR, PDGFR, c-Kit	II	Advanced and RAI-R DTC, MTC and ATC	30%	18.1
Motesanib (Sherman et al. [87])	VEGFR, PDGFR, c-Kit	II	RAI-R DTC	14%	9.3
Sunitinib (Carr et al. [88])	PDGFR. FLT3, c-Kit, VEGFR, RET	II	RAI-R DTC and MTC	31%	12.8
Pazopanib (Bible et al. [89])	VEGFR, PDGFR, c-Kit	II	RAI-R DTC	49%	11.7
Dovitinib (Lim et al. [90])	FGFR, VEGFR	II	Metastatic DTC and MTC	20.5%	5.4
Selumetinib (Hayes et al. [91])	MEK-1/2 (one of MAPK), RAS, BRAF	II	RAI-R DTC	3%	8
Cabozantinib (Cabanillas et al. [92])	VEGFR, RET, MET	I	Advanced DTC	53%	NR
Cabozantinib (Cabanillas et al. [93])	VEGFR, RET, MET	II	RAI-R DTC	40%	12.7
Cabozantinib (Brose et al. [94])	VEGFR, RET, MET	II	RAI-R DTC, Advanced DTC	54%	NR
Sorafenib (Schneider et al. [95])	VEGFR, PDGFR, BRAF	II	RAI-R DTC	31%	18
Vandetanib (Leuboulleux et al. [96])	VEGFR, EGFR, RET	II	RAI-R DTC	8.3%	11.1
Dabrafenib (Falchook et al. [97])	BRAF	I	BRAF-positive advanced thyroid cancer	29%	11.3
Vemurafenib (Brose et al. [98])	BRAF	II	BRAF-positive RAI-R PTC	35%	15.6

Abbreviations: ATC, anaplastic thyroid cancer; DTC, differentiated thyroid cancer; MTC, medullary thyroid cancer; PTC, papillary thyroid cancer; RAI-R, radioiodine-refractory.

## Abbreviations

RTK	receptor tyrosine kinase
VEGFR	vascular endothelial growth factor receptor
PDGFR	platelet-derived growth factor receptor
RET	rearranged during transfection
HER	human epidermal growth factor receptor
PI3K	phosphoinositide 3-kinase
PTEN	phosphatase and tensin homolog
AKT	protein kinase B
mTOR	mechanistic target of rapamycin
RAS	rat sarcoma
RAF	rapidly accelerated fibrosarcoma
MAPK	mitogen-activated protein kinase
ERK	extracellular signal-regulated kinase
PAX8	paired box gene 8
TSH	thyroid stimulating hormone
TSHR	thyroid stimulating hormone receptor
PTTG1	pituitary tumor transforming gene 1
PBF	PTTG1 binding factor
PIGU	phosphatidylinositol glycan anchor biosynthesis class U
NIS	Sodium/Iodide symporter
GPI	glycosylphosphatidylinositol
PKA	protein kinase A
cAMP	cyclic adenosine monophosphate

## References

[1] Haugen B.R., Alexander E.K., Bible K.C., Doherty G.M., Mandel S.J., Nikiforov Y.E., Pacini F., Randolph G.W., Sawka A.M., Schlumberger M. (2016). 2015 American Thyroid Association Management Guidelines for Adult Patients with Thyroid Nodules and Differentiated Thyroid Cancer: The American Thyroid Association Guidelines Task Force on Thyroid Nodules and Differentiated Thyroid Cancer. Thyroid.

[2] Zarnegar R., Brunaud L., Kanauchi H., Wong M., Fung M., Ginzinger D., Duh Q., Clark O.H. (2002). Increasing the effectiveness of radioactive iodine therapy in the treatment of thyroid cancer using Trichostatin A, a histone deacetylase inhibitor. Surgery.

[3] Worden F. (2014). Treatment strategies for radioactive iodine-refractory differentiated thyroid cancer. Ther. Adv. Med. Oncol..

[4] Xing M., Haugen B.R., Schlumberger M. (2013). Progress in molecular-based management of differentiated thyroid cancer. Lancet.

[5] Nixon I.J., Whitcher M.M., Palmer F.L., Tuttle R.M., Shaha A.R., Shah J.P., Patel S.G., Ganly I. (2012). The Impact of Distant Metastases at Presentation on Prognosis in Patients with Differentiated Carcinoma of the Thyroid Gland. Thyroid.

[6] Durante C., Haddy N., Baudin E., Leboulleux S., Hartl D., Travagli J.P., Caillou B., Ricard M., Lumbroso J.D., De Vathaire F. (2006). Long-Term Outcome of 444 Patients with Distant Metastases from Papillary and Follicular Thyroid Carcinoma: Benefits and Limits of Radioiodine Therapy. J. Clin. Endocrinol. Metab..

[7] Gillanders S.L., O’Neill J.P. (2018). Prognostic markers in well differentiated papillary and follicular thyroid cancer (WDTC). Eur. J. Surg. Oncol..

[8] Ho A.L., Grewal R.K., Leboeuf R., Sherman E.J., Pfister D.G., Deandreis D., Pentlow K.S., Zanzonico P.B., Haque S., Gavane S. (2013). Selumetinib-Enhanced Radioiodine Uptake in Advanced Thyroid Cancer. N. Engl. J. Med..

[9] Brose M.S., Nutting C.M., Jarzab B., Elisei R., Siena S., Bastholt L., de la Fouchardiere C., Pacini F., Paschke R., Shong Y.K. (2014). Sorafenib in radioactive iodine-refractory, locally advanced or metastatic differentiated thyroid cancer: A randomised, double-blind, phase 3 trial. Lancet.

[10] Schlumberger M., Tahara M., Wirth L.J., Robinson B., Brose M.S., Elisei R., Habra M.A., Newbold K., Shah M.H., Hoff A.O. (2015). Lenvatinib versus Placebo in Radioiodine-Refractory Thyroid Cancer. N. Engl. J. Med..

[11] Tuttle R.M., Haddad R.I., Ball D.W., Byrd D., Dickson P., Duh Q., Ehya H., Haymart M., Hoh C., Hunt J.P. (2014). Thyroid Carcinoma, Version 2.2014. J. Natl. Compr. Cancer Netw. JNCCN.

[12] Vaisman F., Carvalho D.P., Vaisman M. (2015). A new appraisal of iodine refractory thyroid cancer. Endocr. Relat. Cancer.

[13] Capdevila J., Galofré J., Grande E., Zafón Llopis C., Ramón y Cajal Asensio T., Navarro González E., Jiménez-Fonseca P., Santamaría Sandi J., Gómez Sáez J., Riesco Eizaguirre G. (2017). Consensus on the management of advanced radioactive iodine-refractory differentiated thyroid cancer on behalf of the Spanish Society of Endocrinology Thyroid Cancer Working Group (GTSEEN) and Spanish Rare Cancer Working Group (GETHI). Clin. Transl. Oncol..

[14] Tuttle R.M., Ahuja S., Avram A.M., Bernet V.J., Bourguet P., Daniels G.H., Dillehay G., Draganescu C., Flux G., Führer D. (2019). Controversies, Consensus, and Collaboration in the Use of 131I Therapy in Differentiated Thyroid Cancer: A Joint Statement from the American Thyroid Association, the European Association of Nuclear Medicine, the Society of Nuclear Medicine and Molecular Imaging, and the European Thyroid Association. Thyroid.

[15] Shen X., Liu R., Xing M. (2017). A six-genotype genetic prognostic model for papillary thyroid cancer. Endocr. Relat. Cancer.

[16] Ravera S., Reyna-Neyra A., Ferrandino G., Amzel L.M., Carrasco N. (2017). The Sodium Iodide Symporter (NIS): Molecular Physiology and Preclinical and Clinical Applications. Annu. Rev. Physiol..

[17] Paladino S., Melillo R.M. (2017). Editorial: Novel Mechanism of Radioactive Iodine Refractivity in Thyroid Cancer. J. Natl. Cancer Inst..

[18] Ohno M., Zannini M., Levy O., Carrasco N., Di Lauro R. (1999). The Paired-Domain Transcription Factor Pax8 Binds to the Upstream Enhancer of the Rat Sodium/Iodide Symporter Gene and Participates in Both Thyroid-Specific and Cyclic-AMP-Dependent Transcription. Mol. Cell. Biol..

[19] Na’ara S., Mahameed K., Amit M., Cohen J.T., Weiler-Sagie M., Albitskiy I., Gil Z., Billan S. (2019). Efficacy of posttreatment radioiodine scanning in patients with differentiated thyroid cancer. Head Neck.

[20] Dohán O., Baloch Z., Bánrévi Z., Livolsi V., Carrasco N. (2001). Rapid Communication: Predominant Intracellular Overexpression of the Na+/I− Symporter (NIS) in a Large Sampling of Thyroid Cancer Cases. J. Clin. Endocrinol. Metab..

[21] Agrawal N., Akbani R., Aksoy B., Ally A., Arachchi H., Asa S., Auman J., Balasundaram M., Balu S., Baylin S. (2014). Integrated Genomic Characterization of Papillary Thyroid Carcinoma. Cell.

[22] Nikiforova M.N., Nikiforov Y.E. (2011). Molecular genetics and diagnosis of thyroid cancer. Nat. Rev. Endocrinol..

[23] Xing M. (2013). Molecular pathogenesis and mechanisms of thyroid cancer. Nat. Rev. Cancer.

[24] Riesco-Eizaguirre G., Rodríguez I., De la Vieja A., Costamagna E., Carrasco N., Nistal M., Santisteban P. (2009). The BRAFV600E oncogene induces transforming growth factor beta secretion leading to sodium iodide symporter repression and increased malignancy in thyroid cancer. Cancer Res..

[25] Murugan A.K., Qasem E., Al-Hindi H., Shi Y., Alzahrani A.S. (2016). Classical V600E and other non-hotspot BRAF mutations in adult differentiated thyroid cancer. J. Transl. Med..

[26] Costamagna E., García B., Santisteban P. (2004). The Functional Interaction between the Paired Domain Transcription Factor Pax8 and Smad3 Is Involved in Transforming Growth Factor-β Repression of the Sodium/Iodide Symporter Gene. J. Biol. Chem..

[27] Zhang Z., Liu D., Murugan A.K., Liu Z., Xing M. (2014). Histone deacetylation of NIS promoter underlies BRAF V600E-promoted NIS silencing in thyroid cancer. Endocr. Relat. Cancer.

[28] Vaisman F., Tala H., Grewal R., Tuttle R.M. (2011). In Differentiated Thyroid Cancer, an Incomplete Structural Response to Therapy Is Associated with Significantly Worse Clinical Outcomes Than Only an Incomplete Thyroglobulin Response. Thyroid.

[29] Amit M., Rudnicki Y., Binenbaum Y., Trejo-Leider L., Cohen J.T., Gil Z. (2014). Defining the outcome of patients with delayed diagnosis of differentiated thyroid cancer. Laryngoscope.

[30] Tam S., Amit M., Boonsripitayanon M., Busaidy N.L., Cabanillas M.E., Waguespack S.G., Gross N.D., Grubbs E.G., Williams M.D., Lai S.Y. (2018). Effect of Tumor Size and Minimal Extrathyroidal Extension in Patients with Differentiated Thyroid Cancer. Thyroid.

[31] Rondeau G., Fish S., Hann L.E., Fagin J.A., Tuttle R.M. (2011). Ultrasonographically Detected Small Thyroid Bed Nodules Identified After Total Thyroidectomy for Differentiated Thyroid Cancer Seldom Show Clinically Significant Structural Progression. Thyroid.

[32] Guy A., Hirsch D., Shohat T., Bachar G., Tirosh A., Robenshtok E., Shimon I., Benbassat C.A. (2014). Papillary Thyroid Cancer: Factors Involved in Restaging N1 Disease After Total Thyroidectomy and Radioactive Iodine Treatment. J. Clin. Endocrinol. Metab..

[33] Urken M.L., Milas M., Randolph G.W., Tufano R., Bergman D., Bernet V., Brett E.M., Brierley J.D., Cobin R., Doherty G. (2015). Management of recurrent and persistent metastatic lymph nodes in well-differentiated thyroid cancer: A multifactorial decision-making guide for the thyroid cancer care collaborative. Head Neck.

[34] Brose M.S., Smit J., Capdevila J., Elisei R., Nutting C., Pitoia F., Robinson B., Schlumberger M., Shong Y.K., Takami H. (2012). Regional approaches to the management of patients with advanced, radioactive iodine-refractory differentiated thyroid carcinoma. Expert Rev. Anticancer Ther..

[35] Tuttle R.M., Ball D.W., Byrd D., Dilawari R.A., Doherty G.M., Duh Q., Ehya H., Farrar W.B., Haddad R.I., Kandeel F. (2010). Thyroid Carcinoma. J. Natl. Compr. Cancer Netw. JNCCN.

[36] Tam S., Amit M., Boonsripitayanon M., Cabanillas M.E., Busaidy N.L., Gunn G.B., Lai S.Y., Gross N.D., Sturgis E.M., Zafereo M.E. (2017). Adjuvant External Beam Radiotherapy in Locally Advanced Differentiated Thyroid Cancer. JAMA Otolaryngol. Head Neck Surg..

[37] Busaidy N.L., Cabanillas M.E. (2012). Differentiated Thyroid Cancer: Management of Patients with Radioiodine Nonresponsive Disease. J. Thyroid Res..

[38] Schmidt A., Iglesias L., Klain M., Pitoia F., Schlumberger M.J. (2017). Radioactive iodine-refractory differentiated thyroid cancer: An uncommon but challenging situation. Arch. Endocrinol. Metab..

[39] Sabra M.M., Dominguez J.M., Grewal R.K., Larson S.M., Ghossein R.A., Tuttle R.M., Fagin J.A. (2013). Clinical Outcomes and Molecular Profile of Differentiated Thyroid Cancers With Radioiodine-Avid Distant Metastases. J. Clin. Endocrinol. Metab..

[40] Brose M.S., Worden F.P., Newbold K.L., Guo M., Hurria A. (2017). Effect of Age on the Efficacy and Safety of Lenvatinib in Radioiodine-Refractory Differentiated Thyroid Cancer in the Phase III SELECT Trial. J. Clin. Oncol. Off. J. Am. Soc. Clin. Oncol..

[41] Evaluation of Efficacy, Safety of Vandetanib in Patients with Differentiated Thyroid Cancer. https://clinicaltrials.gov/ct2/show/NCT01876784.

[42] Brose M.S., Robinson B., Bermingham C., Puvvada S., Borgman A.E., Krzyzanowska M.K., Capdevila J., Sherman S.I. (2019). A phase 3 (COSMIC-311), randomized, double-blind, placebo-controlled study of cabozantinib in patients with radioiodine (RAI)-refractory differentiated thyroid cancer (DTC) who have progressed after prior VEGFR-targeted therapy. J. Clin. Oncol..

[43] Lorusso L., Pieruzzi L., Biagini A., Sabini E., Valerio L., Giani C., Passannanti P., Pontillo-Contillo B., Battaglia V., Mazzeo S. (2016). Lenvatinib and other tyrosine kinase inhibitors for the treatment of radioiodine refractory, advanced, and progressive thyroid cancer. OncoTargets Ther..

[44] Berdelou A., Borget I., Godbert Y., Nguyen T., Garcia M., Chougnet C.N., Ferru A., Buffet C., Chabre O., Huillard O. (2018). Lenvatinib for the Treatment of Radioiodine-Refractory Thyroid Cancer in Real-Life Practice. Thyroid.

[45] Locati L.D., Piovesan A., Durante C., Bregni M., Castagna M.G., Zovato S., Giusti M., Ibrahim T., Puxeddu E., Fedele G. (2019). Real-world efficacy and safety of lenvatinib: Data from a compassionate use in the treatment of radioactive iodine-refractory differentiated thyroid cancer patients in Italy. Eur. J. Cancer.

[46] Burrows N., Resch J., Cowen R.L., Von Wasielewski R., Hoang-Vu C., West C.M., Williams K.J., Brabant G. (2010). Expression of hypoxia-inducible factor 1α in thyroid carcinomas. Endocr. Relat. Cancer.

[47] Zerilli M., Zito G., Martorana A., Pitrone M., Cabibi D., Cappello F., Giordano C., Rodolico V. (2010). BRAFV600E mutation influences hypoxia-inducible factor-1[alpha] expression levels in papillary thyroid cancer. Mod. Pathol..

[48] Ramirez R., Hsu D., Patel A., Fenton C., Dinauer C., Tuttle R.M., Francis G.L. (2000). Over-expression of hepatocyte growth factor/scatter factor (HGF/SF) and the HGF/SF receptor (cMET) are associated with a high risk of metastasis and recurrence for children and young adults with papillary thyroid carcinoma. Clin. Endocrinol..

[49] Scarpino S., Cancellario d’Alena F., Di Napoli A., Pasquini A., Marzullo A., Ruco L.P. (2004). Increased expression of Met protein is associated with up-regulation of hypoxia inducible factor-1 (HIF-1) in tumour cells in papillary carcinoma of the thyroid. J. Pathol..

[50] Bergers G., Hanahan D. (2008). Modes of resistance to anti-angiogenic therapy. Nat. Rev. Cancer.

[51] Kerbel R., Yu J., Tran J., Man S., Viloria-Petit A., Klement G., Coomber B., Rak J. (2001). Possible Mechanisms of Acquired Resistance to Anti-angiogenic Drugs: Implications for the Use of Combination Therapy Approaches. Cancer Metastasis Rev..

[52] Rabiee S., Nadoushan M.J., Rayeni N.M., Ansari I. (2017). Correlation between human epidermal growth factor receptor 2 oncoprotein expression and some prognostic factors in papillary thyroid carcinoma. Indian J. Pathol. Microbiol..

[53] Montero-Conde C., Ruiz-Llorente S., Dominguez J.M., Knauf J.A., Viale A., Sherman E.J., Ryder M., Ghossein R.A., Rosen N., Fagin J.A. (2013). Relief of Feedback Inhibition of HER3 Transcription by RAF and MEK Inhibitors Attenuates Their Antitumor Effects in BRAF -Mutant Thyroid Carcinomas. Cancer Discov..

[54] Kremser R., Obrist P., Spizzo G., Erler H., Kendler D., Kemmler G., Mikuz G., Ensinger C. (2003). Her2/neu overexpression in differentiated thyroid carcinomas predicts metastatic disease. Virchows Arch..

[55] Awad M.M., Shaw A.T. (2014). ALK inhibitors in non-small cell lung cancer: Crizotinib and beyond. Clin. Adv. Hematol. Oncol. HO.

[56] Piva R., Ambrogio C., Inghirami G., Chiarle R., Voena C. (2008). The anaplastic lymphoma kinase in the pathogenesis of cancer. Nat. Rev. Cancer.

[57] Palmer R.H., Vernersson E., Grabbe C., Hallberg B. (2009). Anaplastic lymphoma kinase: Signalling in development and disease. Biochem. J..

[58] Murugan A.K., Xing M. (2011). Anaplastic Thyroid Cancers Harbor Novel Oncogenic Mutations of the ALK Gene. Cancer Res..

[59] Ji J.H., Oh Y.L., Hong M., Yun J.W., Lee H., Kim D., Ji Y., Kim D., Park W., Shin H. (2015). Identification of Driving ALK Fusion Genes and Genomic Landscape of Medullary Thyroid Cancer. PLoS Genet..

[60] Demeure M., Aziz M., Rosenberg R., Gurley S., Bussey K., Carpten J. (2014). Whole-Genome Sequencing of an Aggressive BRAF Wild-type Papillary Thyroid Cancer Identified EML4–ALK Translocation as a Therapeutic Target. World J. Surg..

[61] Chou A., Fraser S., Toon C., Clarkson A., Sioson L., Farzin M., Cussigh C., Aniss A., O’Neill C., Watson N. (2015). A Detailed Clinicopathologic Study of ALK-translocated Papillary Thyroid Carcinoma. Am. J. Surg. Pathol..

[62] Plantinga T.S., Heinhuis B., Gerrits D., Netea M.G., Joosten L.A.B., Hermus R.M.M., Oyen W.J.G., Schweppe R.E., Haugen B.R., Boerman O.C. (2014). mTOR Inhibition Promotes TTF1-Dependent Redifferentiation and Restores Iodine Uptake in Thyroid Carcinoma Cell Lines. J. Clin. Endocrinol. Metab..

[63] De Souza E.C., Padron A.S., Braga W.M., de Andrade B.M., Vaisman M., Nasciutti L.E., Ferreira A.C., de Carvalho D.P. (2010). MTOR downregulates iodide uptake in thyrocytes. J. Endocrinol..

[64] Lopez-Campistrous A., Adewuyi E.E., Benesch M.G.K., Ko Y.M., Lai R., Thiesen A., Dewald J., Wang P., Chu K., Ghosh S. (2016). PDGFRα Regulates Follicular Cell Differentiation Driving Treatment Resistance and Disease Recurrence in Papillary Thyroid Cancer. EBioMedicine.

[65] Haghpanah V., Fallah P., Tavakoli R., Naderi M., Samimi H., Soleimani M., Larijani B. (2016). Antisense-miR-21 enhances differentiation/apoptosis and reduces cancer stemness state on anaplastic thyroid cancer. Tumor Biol..

[66] Sherman E.J., Dunn L.A., Ho A.L., Baxi S.S., Ghossein R.A., Fury M.G., Haque S., Sima C.S., Cullen G., Fagin J.A. (2017). Phase 2 study evaluating the combination of sorafenib and temsirolimus in the treatment of radioactive iodine-refractory thyroid cancer. Cancer.

[67] Schneider T.C., de Wit D., Links T.P., van Erp N.P., van der Hoeven J.J.M., Gelderblom H., Roozen I.C.F.M., Bos M., Corver W.E., Van Wezel T. (2017). Everolimus in Patients With Advanced Follicular-Derived Thyroid Cancer: Results of a Phase II Clinical Trial. J. Clin. Endocrinol. Metab..

[68] Read M.L., Lewy G.D., Fong J.C., Sharma N., Seed R.I., Smith V.E., Gentilin E., Warfield A., Eggo M.C., Knauf J.A. (2011). Proto-oncogene PBF/PTTG1IP Regulates Thyroid Cell Growth and Represses Radioiodide Treatment. Cancer Res..

[69] Smith V.E., Read M.L., Turnell A.S., Watkins R.J., Watkinson J.C., Lewy G.D., Fong J.C.W., James S.R., Eggo M.C., Boelaert K. (2009). A novel mechanism of sodium iodide symporter repression in differentiated thyroid cancer. J. Cell Sci..

[70] Kogai T., Sajid-Crockett S., Newmarch L.S., Liu Y.Y., Brent G.A. (2008). Phosphoinositide-3-kinase inhibition induces sodium/iodide symporter expression in rat thyroid cells and human papillary thyroid cancer cells. J. Endocrinol..

[71] Amit M., Na’ara S., Francis D., Matanis W., Zolotov S., Eisenhaber B., Eisenhaber F., Weiler Sagie M., Malkin L., Billan S. (2017). Post-translational Regulation of Radioactive Iodine Therapy Response in Papillary Thyroid Carcinoma. J. Natl. Cancer Inst..

[72] Albero A., Lopéz J.E., Torres A., de la Cruz L., Martín T. (2016). Effectiveness of chemotherapy in advanced differentiated thyroid cancer: A systematic review. Endocr. Relat. Cancer.

[73] Terme M., Pernot S., Marcheteau E., Sandoval F., Benhamouda N., Colussi O., Dubreuil O., Carpentier A.F., Tartour E., Taieb J. (2013). VEGFA-VEGFR Pathway Blockade Inhibits Tumor-Induced Regulatory T-cell Proliferation in Colorectal Cancer. Cancer Res..

[74] Angell T.E., Lechner M.G., Jang J.K., Correa A.J., LoPresti J.S., Epstein A.L. (2014). BRAF V600E in papillary thyroid carcinoma is associated with increased programmed death ligand 1 expression and suppressive immune cell infiltration. Thyroid.

[75] Bernet V., Smallridge R. (2014). New therapeutic options for advanced forms of thyroid cancer. Expert Opin. Emerg. Drugs.

[76] Chen J., Ji Q., Cao J., Ji D., Bai C., Lin Y., Pan B., Sun G., Li J., Qi C. (2017). A phase II multicenter trial of the multitargeted kinase inhibitor sulfatinib in advanced medullary thyroid cancer (MTC) and radioiodine (RAI)-refractory differentiated thyroid cancer (DTC). JCO.

[77] Makker V., Rasco D., Vogelzang N.J., Brose M.S., Cohn A.L., Mier J., Di Simone C., Hyman D.M., Stepan D.E., Dutcus C.E. (2019). Lenvatinib plus pembrolizumab in patients with advanced endometrial cancer: An interim analysis of a multicentre, open-label, single-arm, phase 2 trial. Lancet Oncol..

[78] Study of Atezolizumab in Combination With Cobimetinib in Participants With Locally Advanced or Metastatic Solid Tumors. https://clinicaltrials.gov/ct2/show/NCT01988896.

[79] Rothenberg S.M., McFadden D.G., Palmer E.L., Daniels G.H., Wirth L.J. (2015). Redifferentiation of Iodine-Refractory BRAF V600E-Mutant Metastatic Papillary Thyroid Cancer with Dabrafenib. Clin. Cancer Res..

[80] Valerio L., Pieruzzi L., Giani C., Agate L., Bottici V., Lorusso L., Cappagli V., Puleo L., Matrone A., Viola D. (2017). Targeted Therapy in Thyroid Cancer: State of the Art. Clin. Oncol..

[81] Cheng L., Jin Y., Liu M., Ruan M., Chen L. (2017). HER inhibitor promotes BRAF/MEK inhibitor-induced redifferentiation in papillary thyroid cancer harboring BRAFV600E. Oncotarget.

[82] Dabrafenib and Lapatinib Ditosylate in Treating Patients with Refractory Thyroid Cancer That Cannot be Removed by Surgery. https://clinicaltrials.gov/ct2/show/NCT01947023.

[83] Nagarajah J., Le M., Knauf J.A., Ferrandino G., Montero-Conde C., Pillarsetty N., Bolaender A., Irwin C., Krishnamoorthy G.P., Saqcena M. (2016). Sustained ERK inhibition maximizes responses of BrafV600E thyroid cancers to radioiodine. J. Clin. Investig..

[84] Efficacy of MEK (Trametinib) and BRAFV600E (Dabrafenib) Inhibitors with Radioactive Iodine (RAI) for the Treatment of Refractory Metastatic Differentiated Thyroid Cancer (MERAIODE). https://clinicaltrials.gov/ct2/show/NCT03244956.

[85] Locati L.D., Licitra L., Agate L., Ou S.I., Boucher A., Jarzab B., Qin S., Kane M.A., Wirth L.J., Chen C. (2014). Treatment of advanced thyroid cancer with axitinib: Phase 2 study with pharmacokinetic/pharmacodynamic and quality-of-life assessments. Cancer.

[86] Cohen E.E., Rosen L.S., Vokes E.E., Kies M.S., Forastiere A.A., Worden F.P., Kane M.A., Sherman E., Kim S., Bycott P. (2008). Axitinib Is an Active Treatment for All Histologic Subtypes of Advanced Thyroid Cancer: Results From a Phase II Study. J. Clin. Oncol..

[87] Sherman S.I., Wirth L.J., Droz J., Hofmann M., Bastholt L., Martins R.G., Licitra L., Eschenberg M.J., Sun Y., Juan T. (2008). Motesanib Thyroid Cancer Study Group Motesanib Diphosphate in Progressive Differentiated Thyroid Cancer. N. Engl. J. Med..

[88] Carr L.L., Mankoff D.A., Goulart B.H., Eaton K.D., Capell P.T., Kell E.M., Bauman J.E., Martins R.G. (2010). Phase II Study of Daily Sunitinib in FDG-PET–Positive, Iodine-Refractory Differentiated Thyroid Cancer and Metastatic Medullary Carcinoma of the Thyroid with Functional Imaging Correlation. Clin. Cancer Res..

[89] Bible K.C., Suman V.J., Molina J.R., Smallridge R.C., Maples W.J., Menefee M.E., Rubin J., Sideras K., Morris J.C., McIver B. (2010). Efficacy of pazopanib in progressive, radioiodine-refractory, metastatic differentiated thyroid cancers: Results of a phase 2 consortium study. Lancet Oncol..

[90] Lim S.M., Chung W.Y., Nam K.H., Kang S.W., Lim J.Y., Kim H.G., Shin S.H., Sun J.M., Kim S.G., Kim J.H. (2015). An open label, multicenter, phase II study of dovitinib in advanced thyroid cancer. Eur. J. Cancer.

[91] Hayes D.N., Lucas A.S., Tanvetyanon T., Krzyzanowska M.K., Chung C.H., Murphy B.A., Gilbert J., Mehra R., Moore D.T., Sheikh A. (2012). Phase II Efficacy and Pharmacogenomic Study of Selumetinib (AZD6244; ARRY-142886) in Iodine-131 Refractory Papillary Thyroid Carcinoma with or without Follicular Elements. Clin. Cancer Res..

[92] Cabanillas M.E., Brose M.S., Holland J., Ferguson K.C., Sherman S.I. (2014). A Phase I Study of Cabozantinib (XL184) in Patients with Differentiated Thyroid Cancer. Thyroid.

[93] Cabanillas M.E., de Souza J.A., Geyer S., Wirth L.J., Menefee M.E., Liu S.V., Shah K., Wright J., Shah M.H. (2017). Cabozantinib As Salvage Therapy for Patients With Tyrosine Kinase Inhibitor–Refractory Differentiated Thyroid Cancer: Results of a Multicenter Phase II International Thyroid Oncology Group Trial. J. Clin. Oncol..

[94] Brose M.S., Shenoy S., Bhat N., Harlacker A.K., Yurtal R.K., Posey Z.A., Torrente D.M., Grande C., Squillante C.M., Troxel A. (2018). A phase II trial of cabozantinib (CABO) for the treatment of radioiodine (RAI)-refractory differentiated thyroid carcinoma (DTC) in the first-line setting. JCO.

[95] Schneider T.C., Abdulrahman R.M., Corssmit E.P., Morreau H., Smit J.W.A., Kapiteijn E. (2012). Long-term analysis of the efficacy and tolerability of sorafenib in advanced radio-iodine refractory differentiated thyroid carcinoma: Final results of a phase II trial. Eur. J. Endocrinol..

[96] Leboulleux S., Bastholt L., Krause T., de la Fouchardiere C., Tennvall J., Awada A., Gómez J.M., Bonichon F., Leenhardt L., Soufflet C. (2012). Vandetanib in locally advanced or metastatic differentiated thyroid cancer: A randomised, double-blind, phase 2 trial. Lancet Oncol..

[97] Falchook G.S., Millward M., Hong D., Naing A., Piha-Paul S., Waguespack S.G., Cabanillas M.E., Sherman S.I., Ma B., Curtis M. (2015). BRAF Inhibitor Dabrafenib in Patients with Metastatic BRAF-Mutant Thyroid Cancer. Thyroid.

[98] Brose M.S., Cabanillas M.E., Cohen E.E., Wirth L.J., Riehl T., Yue H., Sherman S.I., Sherman E.J. (2016). Vemurafenib in patients with BRAFV600E -positive metastatic or unresectable papillary thyroid cancer refractory to radioactive iodine: A non-randomised, multicentre, open-label, phase 2 trial. Lancet Oncol..

